# Characterization of Episomal Replication of Bovine Papillomavirus Type 1 DNA in Long-Term Virion-Infected *Saccharomyces Cerevisiae* Culture

**DOI:** 10.1007/s12250-021-00439-y

**Published:** 2021-08-30

**Authors:** Quanmei Tu, Weixu Feng, Zhuo Chen, Qijia Li, Yu Zhao, Jun Chen, Pengfei Jiang, Xiangyang Xue, Lifang Zhang, Kong-Nan Zhao

**Affiliations:** 1 Department of Obstetrics and Gynaecology, The Second Affiliated Hospital and Yuyin Children Hospital of Wenzhou Medical University, Wenzhou, 325035 China; 2 Institute of Molecular Virology and Immunology, Department of Microbiology and Immunology, School of Basic Medical Sciences, Wenzhou Medical University, Wenzhou, 325035 China; 3 Australian Institute for Bioengineering and Nanotechnology, the University of Queensland, St Lucia, 4067 Australia

**Keywords:** Bovine papillomavirus type 1 (BPV-1), *Saccharomyces cerevisiae*, Genomic DNA replication, Agarose gel electrophoresis, Southern blot hybridization

## Abstract

We have previously reported that bovine papillomavirus type 1 (BPV-1) DNA can replicate its genome and produce infectious virus-like particles in short term virion-infected *S. cerevisiae* (budding yeast) cultures (Zhao and Frazer 2002, Journal of Virology, 76:3359–64 and 76:12265–73). Here, we report the episomal replications of BPV-1 DNA in long term virion-infected *S. cerevisiae* culture up to 108 days. Episomal replications of the BPV-1 DNA could be divided into three patterns at three stages, early active replication (day 3–16), middle weak replication (day 23–34/45) and late stable replication (day 45–82). Two-dimensional gel electrophoresis analysis and Southern blot hybridization have revealed further that multiple replication intermediates of BPV-1 DNA including linear form, stranded DNA, monomers and higher oligomers were detected in the virion-infected yeast cells over the time course. Higher oligomers shown as covalently closed circular DNAs (cccDNAs) are the most important replication intermediates that serve as the main nuclear transcription template for producing all viral RNAs in the viral life cycle. In this study, the cccDNAs were generated at the early active replication stage with the highest frequencies and then at late stable replication, but they appeared to be suppressed at the middle weak replication. Our data provided a novel insight that BPV-1 genomic DNA could replicate episomally for the long period and produce the key replication intermediates cccDNAs in *S. cerevisiae* system.

**Supplementary Information:**

The online version contains supplementary material available at 10.1007/s12250-021-00439-y.

## Introduction

Papillomaviruses (PVs) are a family of small double-stranded circular DNA viruses with more than 200 genotypes (Doorbar 2016). Human papillomaviruses (HPVs) have been demonstrated to be responsible for causing several human cancers and genital warts (Moody and Laimins, 2010). The persistent infection of high-risk human papillomavirus (hr-HPV) is closely related to the pathogenesis of cervical cancer and many other cancers. Indeed, HPVs account for more than 30% of all infection-associated cancers in humans (zur Hausen 2009). Preventive vaccines including 2-valent, 4-valent and 9-valent vaccines against HPV-associated cervical cancer are now available, which prevent an estimated 92% of the cancers attributable to HPV types (Frazer and Levin 2011; Huh *et al.* 2017; Zhang* et al.* 2020a). Considering that the preventive vaccines are unable to wipe out the existing HPV viruses in already infected people, scientists have been focusing on developing therapeutic HPV vaccines that have reached the clinical trial phase in cervical cancers and diseases. Many therapeutic HPV vaccines tested in clinical trials show the potential use of the vaccines as safe and effective pharmacological tools (Ding* et al.* 2010; Zhang* et al*. 2020a; Zhao* et al.* 1998; Zottnick* et al.* 2020). Recently, published studies have revealed that HPV minor capsid L2 proteins play a crucial role in the early stage of HPV infection (DiGiuseppe* et al.* 2015; Gagnon *et al.* 2015; Zhang* et al.* 2018, 2020b). The C terminus of HPV L2 proteins contains a short sequence of basic amino acids serving as a cell-penetrating peptide (CPP) (Campos 2017; DiGiuseppe* et al.* 2015; Zhang* et al.* 2018). The CPP binds to a cytoplasmic trafficking factor, retromer, thereby sorting the virus into the retrograde pathway for properly trafficking the incoming virus to the nucleus, the site of viral gene expression and DNA replication (Campos 2017; DiGiuseppe* et al.* 2015; Zhang* et al.* 2018). Thus, it appears that there is a need for specific drugs for targeting the CPP of L2 proteins to prevent HPV infection potentially leading to the development of HPV therapy against HPV-induced cancer.

PVs have also been identified in most domestic animals such as bovines (BPVs), canines (CPVs), goats (*Capra hircus* papillomavirus-ChPVs), equines (*Eqnus caballus* papillomavirus EcPVs) (Modolo* et al.* 2017). BPVs are the most typical animal PVs, which are a cosmopolitan virus, worldwide distributed, independently of the level of expertise on livestock exploration (Araldi* et al.* 2017). BPVs containing 15 types have been classified into 3 separate genera: *Deltapapillomavirus*, *Epsilonpapillomavirus*, and *Xipapillomavirus*, which are causative agents of benign and malignant tumors in cattle, such as cutaneous papilloma, fibropapilloma, urinary bladder and esophageal cancers (Borzacchiello and Roperto 2008; Campo 2003; Munday* et al.* 2015). BPV-1, BPV-2, and BPV-13, for example, are classified in the *Deltapapillomavirus* genus and induce fibropapilloma (Munday* et al.* 2015; Thomson* et al.* 2015). Since 1970, BPV-1 has been used as a prototype papillomavirus to study PV biology and oncology (Araldi* et al.* 2017; Borzacchiello and Roperto 2008; Borzacchiello* et al.* 2006; Koller and Olson 1972). As a model system, BPV-1 has been used to study the regulation of DNA replication in higher eukaryotes (Schvartzman* et al.* 1990). In addition, transcription of BPV-1 viral RNAs has been previously reported in BPV-1 transformed cells and in warts (Amtmann and Sauer, 1982). The study of using BPV has greatly improved our understanding of HPV-associated carcinogenesis (Munday* et al.* 2015). We and others have previously developed an *in vitro* *Saccharomyces cerevisiae* (budding yeast) system, which is permissive for viral DNA replication (e.g. BPV-1 and different types of HPVs) (Angeletti* et al.* 2002; Zhao and Frazer 2002a, b). We discovered that *S. cerevisiae* was infected with BPV-1 virions isolated from the bovine papilloma leading to not only the replication of BPV-1 genomic DNA, but also the production of infectious virus-like particles (Zhao and Frazer 2002a, b). The use of *S. cerevisiae* for the replication of both plant and animal viruses was a breakthrough (Navarro* et al.* 2004).

Several studies have reported the replication patterns of PV genome in different systems by means of different techniques such as Southern blot, chromogenic *in situ* hybridization (CISH) and PCR using specific and/or degenerate primers (Araldi* et al.* 2015; Melo CA and Melo SA, 2014; Melo* et al.* 2015). In addition, using the restriction fragment length polymorphism of PCR products (PCR-RFLP) allows to identify BPV type (Carvalho* et al.* 2013), since this method shows a correlation of 95% with the results obtained using DNA sequencing (Carvalho* et al.* 2013; Kawauchi* et al.* 2015). However, the above methods cannot be used to characterize the replication mode and intermediates of the viral DNA that persists as extrachromosomal plasmids in eukaryotic cells and neither to resolve the initiation, elongation, and termination of DNA replication associated with distinct, nonlinear DNA structures. Bell and Byers first developed two-dimensional agarose gel electrophoresis (2-DAGE) to study the shape of DNA recombination intermediates (Bell and Byers 1983). The 2-DAGE includes a first and a second (run perpendicular to the first) dimension agarose gel electrophoresis. The first dimension is a conventional separation of DNA by molecular size. The second dimension is to separate the molecules mainly on the basis of the DNA shape. Since then, 2-DAGE has been proved to be highly advanced and useful for studying the complex topological problem of DNA replication. The rationale and advances of 2-DAGE for DNA replication study include: (1) mapping origins and termination sites of DNA replication; (2) investigating the efficiency of different origins and the progress of DNA replication forks along DNA fragments; (3) determining the activity level of putative replication origin-containing sequences; (4) analyzing replication timing, fork progression, fork pausing, fork stalling and collapse, termination, and recombinational repair; and (5) resolving the nonlinear, replicating DNA molecules from the linear, nonreplicating molecules (Dandjinou* et al.* 2006; Lemacon* et al.* 2017; Makovets 2013; Olavarrieta* et al.* 2002; Quinet* et al.* 2017). Indeed, Schvartzman *et* *al.* identified extrachromosomal forms of BPV-1 DNA replication in viral DNA-transformed ID13 cells by 2-DAGE (Schvartzman* et al.* 1990). We have also observed a single replication bubble of BPV-1 DNA in short-term virion-infected *S. cerevisiae* cultures by 2-DAGE (Zhao and Frazer 2002a).

Although multiple HPV genomes replicate stably in *S. cerevisiae* for a long-term (up to 75 h), but they differ in replication efficiency (Rogers* et al.* 2008). The patterns of PV DNA replication and the replication intermediates in *S. cerevisiae* remain unknown. Thus, we proposed to study the episomal replication patterns and intermediates of BPV-1 DNA in long-term virion-infected *S. cerevisiae* cultures (up to 108 days) by means of one- and two-dimensional gel electrophoresis and Southern blot hybridization. As an attempt to use BPV-1 model, we studied the regulation of DNA replication in a latently virion-infected single-celled eukaryotic organism. The obtained data have highlighted the characteristics of BPV-1 genomic DNA giving some insights of virus infection in *S. cerevisiae* system although the exact mechanism of BPV-1 DNA replication remains unclear.

## Materials and Methods

### BPV-1 Virion Preparation

BPV-1 virions used for *S. cerevisiae* protoplast infection were prepared from bovine papilloma as reported previously (Zhao and Frazer 2002a, b). Virions in suspension were dialyzed against 0.15 mol/L phosphate-buffered saline (PBS) (pH 7.4) for 30 min and then used to infect *S. cerevisiae* protoplasts.

### Cultures of BPV-1-Infected *S. cerevisiae* Cells and Sample Collection

BPV-1-infected *S. cerevisiae* cells were grown *in vitro* for a long-term culture, similar to our previous description (Zhao and Frazer 2002a, b), with some modifications. Briefly, *S. cerevisiae* protoplasts (10 mL; 5 × 10^7^ cells/mL) infected with 0.6 µg of BPV-1 virions was grown in *S. cerevisiae* medium containing 0.8 mol/L sorbitol and 0.2 mol/L glucose on a shaker with gentle agitation at 28 °C in the dark for three separate long time courses (82, 85 and 108 days). *S. cerevisiae* protoplasts without infection of BPV-1 virions were cultured under the same condition as a negative control. Ten to twelve time points were designed to collect *S. cerevisiae* cells for analysis of viral DNA replication. At each time point, 5 mL of *S. cerevisiae* cells was collected and allocated for Hirt supernatant DNA (Hirt DNA), RNA and protein preparation, with 5 mL of fresh medium without sorbitol added for continuous culture.

### Hirt DNA Preparation

BPV-1-infected *S. cerevisiae* cultures were used for the preparation of Hirt DNA as described. Digestion of *S. cerevisiae* cells with enzyme was the same as that for *S. cerevisiae* protoplast preparation. The digested *S. cerevisiae* cells were washed with 1 mol/L sorbitol and lysed in 400 µL of lysate buffer (10 mmol/L Tris–HCl, pH 7.5, 10 mmol/L EDTA, and 0.2% Triton X-100) at 25 °C for 10 min. Then 100 µL of 5 mol/L NaCl was added to the lysate, and the mixture was frozen at −20 °C for 40 min. The frozen lysates were thawed at 25 °C for 20 min. The resultant supernatants containing viral DNA (Hirt DNA) were incubated with 100 µg of proteinase K at 37 °C for 1 h. The Hirt DNA was extracted with Tris-buffered phenol twice and chloroform once and then precipitated by ethanol. The Hirt DNA was then used for agarose gel electrophoresis and Southern blot hybridization**.**

### One-Dimensional Agarose Gel Electrophoresis and Southern Blot Hybridization

Hirt DNA (10 µg) partially digested with Hind III as previously reported (Zhao and Frazer 2002a, b) was electrophoresed on a 1% one-dimensional agarose gel electrophoresis (1-DAGE), then blotted onto nylon membranes, and hybridized with a ^32^P-labeled BPV-1 DNA probe (Zhao and Frazer 2002a).

### Two-Dimensional Agarose Gel Electrophoresis and Southern Blot Hybridization

Hirt DNA (15 µg) without enzyme digestion as reported by Schvartzman and colleagues (Schvartzman* et al.* 1990) was separated on a neutral/alkaline 2-DAGE. The Hirt DNA was electrophoresed on a first-dimension agarose gel (0.4%) in TAE buffer (40 mmol/L Tris-acetate, 2 mmol/L EDTA, pH 8.0) at 1.5 V/cm for 20 h. The DNA lanes were run on a second-dimension agarose gel (1.0% agarose in sterile distilled H_2_O) in alkaline electrophoresis buffer (40 mmol/L NaOH, 2 mmol/L EDTA) at 1.5 V/cm for 24 h and then blotted onto nylon membranes. The DNA blots were hybridized with a ^32^P-labeled BPV-1 DNA probe that was a linearized BPV genome to detect all the replication intermediates (Zhao and Frazer 2002a, b). According to previous studies (Flores and Lambert 1997; Sakakibara* et al.* 2013; Schvartzman* et al.* 1990), the replication mode and intermediates of the BPV-1 DNA in *S. cerevisiae* were analyzed and identified. The replication intermediates included single-stranded DNA (1ss), double-stranded DNA (2ss), heterogeneous double-stranded DNA (hds), linear forms (lin) and a series of oligomers. Replication intermediates also included recombinant-dependent replication (RDR), rolling circle replication (RCR) and conspicuous multimeric circular ssDNA (cms). A diagram drawn from the visualized Southern blot hybridization results was used to explain in details the identified replication intermediates of BPV-1 DNA occurred in *S. cerevisiae* cells in Result section.

## Results

### Episomal Replication of BPV-1 DNA in Long-Term Virion-Infected *S. cerevisiae* Cultures

**Fig. 1:**
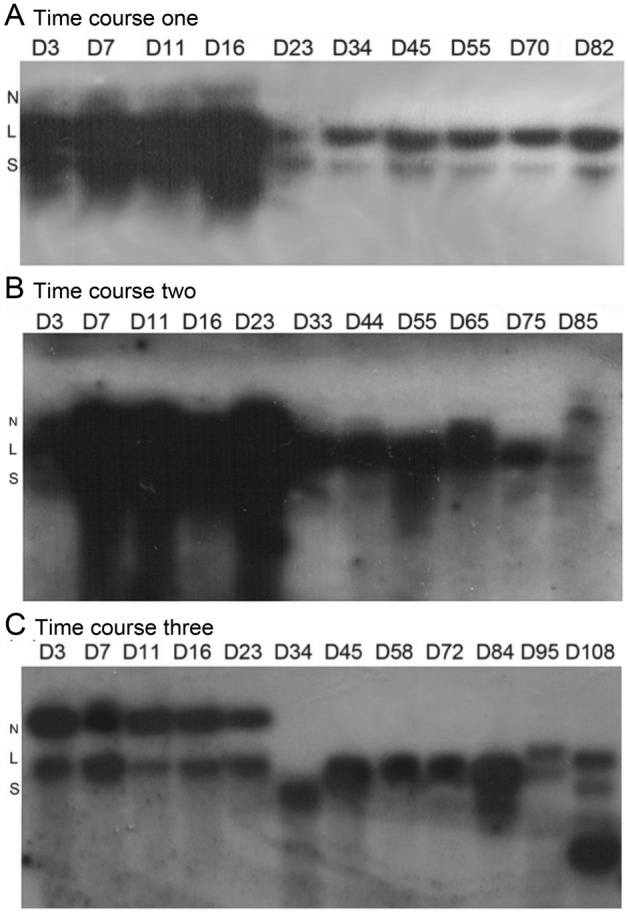
BPV-1 DNA detected by Southern blot hybridization in BPV-1 virion-infected *S. cerevisiae* culture in three-time course experiments: day 0 to day 82 (**A**), day 0 to day 85 (**B**), day 0 to day 108 (**C**). *S. cerevisiae* cells (10 mL; 5 × 10^7^cells/mL) infected with 0.6 µg of BPV-1 was cultured at 28 °C in the dark. At each time point, 5 mL of *S. cerevisiae* cells was collected for Hirt DNA preparation, with 5 mL of fresh medium added for continuous culture. 10 µg of Hirt DNA without partial digestion of *Hin*dIII was electrophoresed on a 1% agarose gel and blotted onto a nylon membrane. Blots were hybridized with BPV-1 DNA using [α-^32^P] dCTP at 3000 Ci/mmol and exposed using Kodak BioMax film at −70 °C for 24 h. N, nicked circular BPV-1 DNA; L, linear BPV-1 DNA; S, supercoiled monomeric BPV-1 DNA

We previously demonstrated that BPV-1 DNA could be detected in a small volume (2 mL) of *S. cerevisiae* cells infected with BPV-1virions at day 55 and 75 post-infection by 1-DAGE and Southern blot hybridization of Hirt DNA (Zhao and Frazer 2002a, b). Thus, to confirm further that BPV-1 DNA could be persistently present in the BPV-1-infected *S. cerevisiae* cells in a relatively large volume (10 mL) cultured for a longer period at 28 °C, we carried out three separate long term BPV-1 virion-infected *S. cerevisiae* culture experiments up to 108 days. It was observed that the BPV-1-infected *S. cerevisiae* cells grew slowly from day 23/34 to day 45 and most of the cells showed unhealthy morphology (Supplementary Fig. S1). We prepared Hirt DNA from the long term BPV-1-infected *S. cerevisiae* cultures at various time points to investigate whether and how BPV-1 DNA was present by Southern blot analysis. The Hirt DNA was subjected to partial digestion with *Hin*dIII, which should cut BPV-1 DNA at a single site. The partial-digested Hirt DNA hybridized with BPV-1 DNA revealed that BPV-1 DNA was detected in all three long-term BPV-1-infected *S. cerevisiae* cultures (Fig. 1). The present forms of BPV-1 DNA in the long-term BPV-1-infected *S. cerevisiae* cultures varied distinctly from the early stage to the late stage (Fig. 1). At the early stage (day 3 to day 16/23), three bands of the BPV-1 DNA were observed to correspond to nicked circular, linear, and supercoiled monomeric BPV-1 DNA although the signals of nicked circular form were very weak. At the middle and late stages (day 23 to day 82), nicked circular form was undetectable, while signals of the other two forms decreased significantly at day 23/34 then at a constant level. Compared with the nicked circular and supercoiled monomeric forms, the linear BPV-1 DNA had the strongest signals over the time period (Fig. 1A), which could be attributed to the partial digestion of *Hin*dIII. The present patterns of BPV-1 DNA detected in the three long-term BPV-1-infected *S. cerevisiae* cell cultures were generally similar, but the signals of nicked circular and supercoiled forms differed (Fig. 1). The results confirmed that *S. cerevisiae* infected with BPV-1 virions could maintain episomal BPV-1 DNA for a long-term period. The results also suggested that the viral genome was actively amplified in the BPV-1-infected *S. cerevisiae* cells at the early stage, and then replicated at a low and constant copy number at the middle and late stages to establish persistent infection in the *S. cerevisiae* cells*.*

### DNA Replication Intermediates in Long-Term BPV-1-Infected *S. cerevisiae* Cultures

**Fig. 2:**
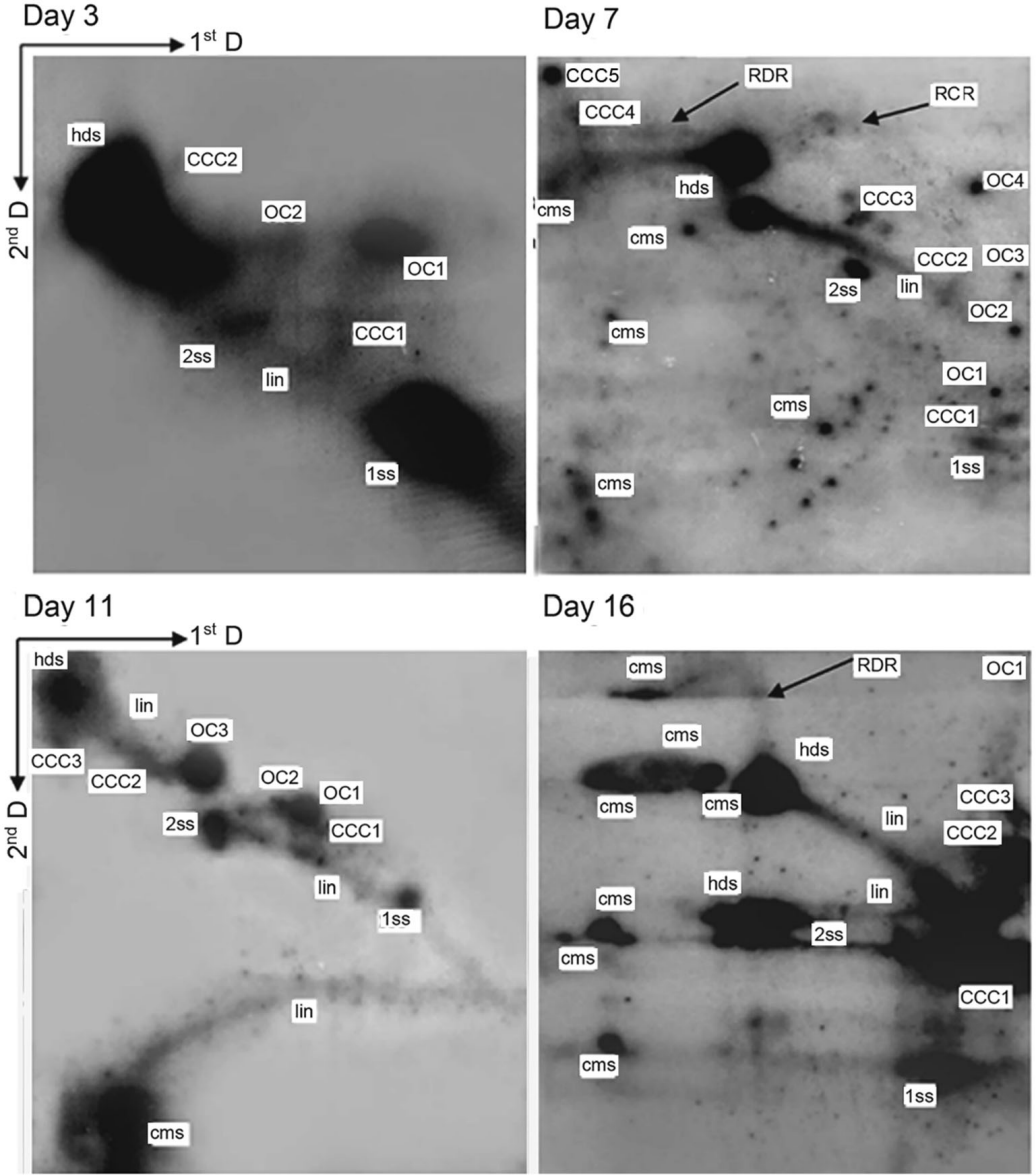
Analysis of viral DNA replication intermediates in BPV-1 virion-infected *S. cerevisiae* cells culture for 3, 7, 11 and 16 days by means of two-dimensional gel electrophoresis. 15 µg Hirt DNA was electrophoresed on a first-dimension agarose gel (0.4%) in TAE buffer (40 mmol/L Tris–acetate, 2 mmol/L EDTA, pH 8.0) at 1.5 V/cm for 20 h. The DNA lanes were run on a second-dimension agarose gel (1.0% agarose in sterile distilled H_2_O) in alkaline electrophoresis buffer (40 mmol/L NaOH, 2 mmol/L EDTA) at 1.5 V/cm for 24 h and then blotted onto nylon membranes. The DNA blots were hybridized with a ^32^P-labeled BPV-1 DNA probe. Replication intermediates include single-stranded DNA (1ss), double-stranded DNA (2ss), heterogeneous double-stranded DNA (hds), linear forms (lin) and a series of oligomers. The oligomers contain monomers (1), dimers (2), trimers (3), and higher oligomers (4 or 5), which showed as covalently closed circles (CCC) and open circles (OC). Replication intermediates also include conspicuous multimeric circular ssDNA (cms), rolling circle replication (RCR) and recombinant-dependent replication (RDR) intermediates.

**Fig. 3:**
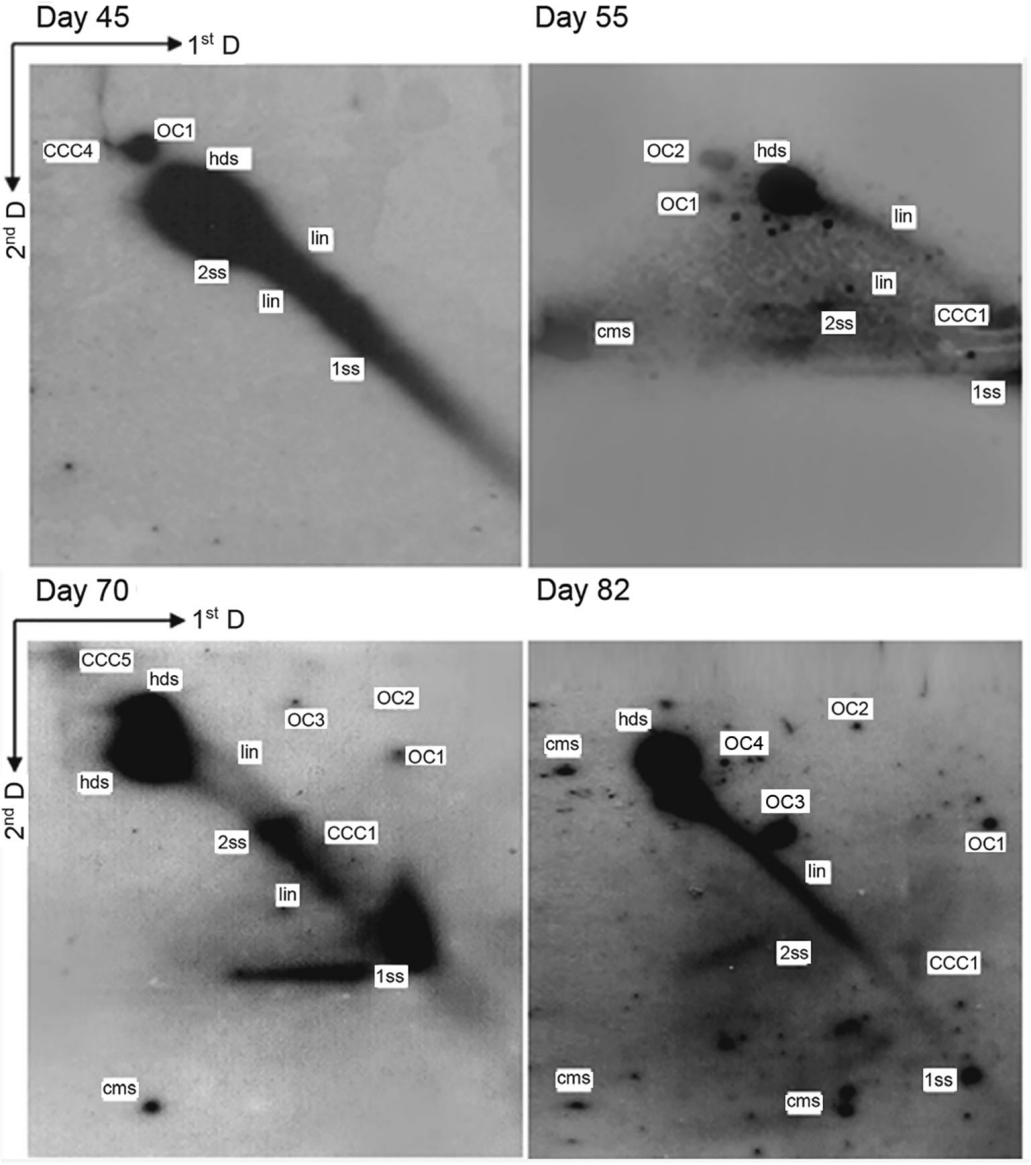
Analysis of viral DNA replication intermediates in BPV-1 virion-infected *S. cerevisiae* cells culture for 45, 55, 70 and 82 days by means of two-dimensional gel electrophoresis and Southern blot-hybridization analysis. Replication intermediates were detected including not only single-stranded DNA (1ss), double-stranded DNA (2ss), heterogeneous double-stranded DNA (hds), linear forms (lin) but also a series of oligomers such as covalently closed circles (CCC), open circles (OC), conspicuous multimeric circular.

We have previously confirmed that BPV-1 DNA detected in the short term BPV-1-infected *S. cerevisiae* culture at one-time point was replicating, with a single replication bubble observed, which was a typical replication pattern by 2-DAGE and Southern blot hybridization (Zhao and Frazer 2002a). Here, the Hirt DNA samples without *Hin*dIII digestion were used to carry out 2-DAGE and Southern blot hybridization to analyze newly synthesized viral genomic DNA. Most of the newly replicated duplex DNA was converted into small molecules: the replication intermediates, which served as the templates for additional replication or transcription. The generation of the replication intermediates of viral DNA over the time course could be divided into three culture stages: early (day 3–16), middle (day 23–34/45), and late-stage (day 45–82). At the early culture stage (day 3, 7, 11 and 16), much more multiple replication intermediates such as the linear monomers, dimers, trimers, and higher oligomers were generated and shown as covalently closed circles (CCC1, CCC2, CCC3, CCC4 and CCC5) and open circles (OC1, OC2, OC3, OC4) (Fig. 2). The cccDNAs are the most important replication intermediates that serve as the main nuclear transcription template for producing all viral RNAs in the viral life cycle. Furthermore, the replication intermediates including 1ss, 2ss and lin consistently occurred at this stage (Fig. 2). Also, other replication intermediates such as hds, cms, RCR and RDR intermediates were detected (Fig. 2). Although so many different replication intermediates were detected, the occurrences of these intermediates except for 1ss, 2ss and lin forms were significantly different from one-time point to another time point. Generally, significantly more replication intermediates were detected in the early culture stage (day 3, 7, 11 and 16) than those in the middle culture stage (day 23 and 34) (Supplementary Fig. S2) and late culture stage (day 45, 55, 72 and 82) (Fig. 3). In fact, only 1ss, 2ss, lin and hds forms were present, other replication intermediates were scarcely detected in the middle culture stage (day 23 and 34) (Supplementary Fig. S2). It appeared that much less replication intermediates occurred in the middle stage were associated with poor growth of the BPV-1-infected *S. cerevisiae* cells (Supplementary Fig. S1) and poor replication status of the viral DNA (Fig. 1). In the late culture stage, replication intermediates such as CCC1, CCC5, OC1, OC2, OC3, OC4 and cms could be detected, but not as many as those observed at the early culture stage (Figs. 2 and 3). In addition, Y-shaped replication intermediates were observed at several time points in both early and late stages (Figs. 2 and 3).

### Diagrammatic Explanation of BPV-1 DNA Replication Intermediates in *S. cerevisiae*

**Fig. 4:**
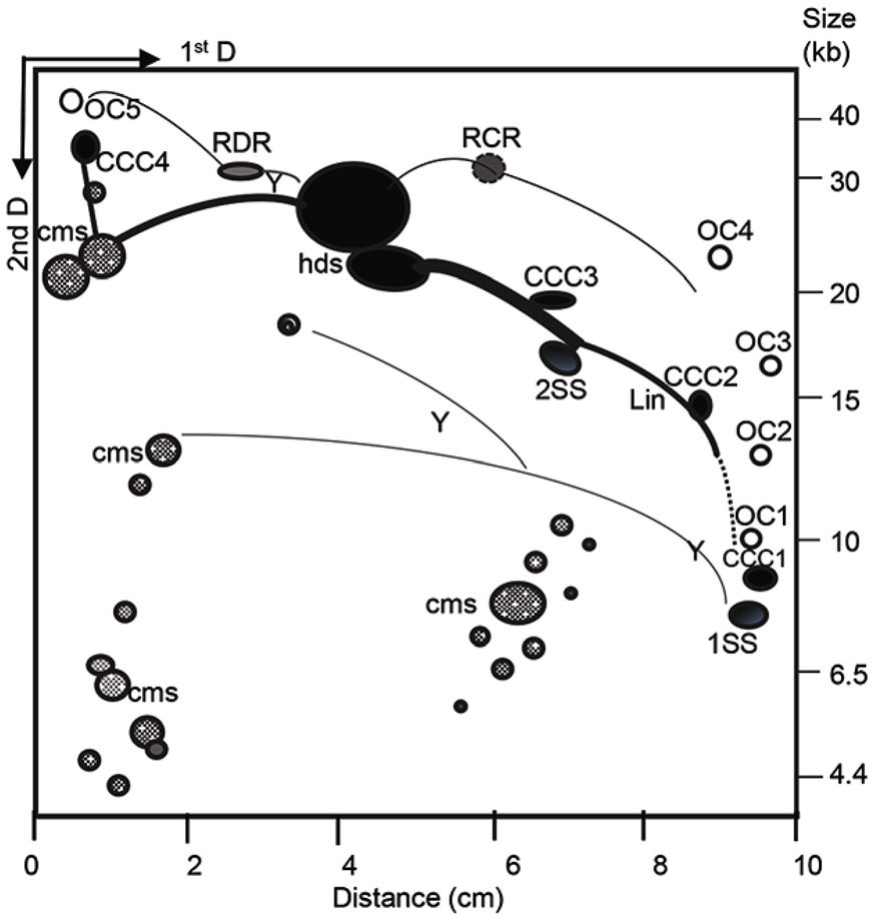
Diagram of replication intermediates of BPV-1 DNA in BPV-1 virion-infected *S. cerevisiae* cells culture mainly based on the Day-7 visualized 2-DAGE/Southern blotting result (Fig. 2). The replication intermediates include single-stranded DNA (1ss), double-stranded DNA (2ss), heterogeneous double-stranded DNA (hds), linear forms (lin). The replication intermediates also include multiple oligomers that are covalently closed circles (CCC), open circles (OC), recombinant-dependent replication (RDR), rolling circle replication (RCR) and conspicuous multimeric circular ssDNA (cms). In addition, 2.3-kb viral DNA fragment would be replicated by a single fork traversing the fragment (Y) and by two converging forks (Y) that met at the termination site for DNA replication.

We have drawn a diagram to explain the identified replication intermediates of BPV-1 DNA in *S. cerevisiae* cells mainly based on the day-7 visualized 2-DAGE/Southern blotting result (Fig. 4). Firstly, the identified replication intermediates included 1ss, 2ss, hds, lin and a series of oligomers. The size of 1ss was about 8 kb while the 2ss was approximately 16 kb and hds was over 23 kb. The identified oligomers contained monomers, dimmers, trimers, and higher oligomers, which showed as CCC and OC. Both CCC and OC were detected above the arc of linear molecules, with their occurrences in different sizes associated with the arc numbers of linear molecules. Generally, the OC has shown a slightly higher molecular size than CCC within an arc of linear molecules. Then, the identified replication intermediates included RDR, RCR and cms. RDR is one of HPV DNA replication modes (Sakakibara* et al.* 2013). RDR occurred in a high molecular size is recognized as important for replication restart and stability, which plays an essential role in the replication cycle of HPV DNA. RCR is a single nicking event on one parental DNA strand, which gives rise to unidirectional replication resulting from the convergence of two replication forks without forming intermediates and termination structures (Flores and Lambert 1997). cms was detected under the arc of linear molecules and generated from the oligomerization of circular single-stranded DNA. cms is independent on the topology of the input DNA and has various molecular sizes due to rapid binding of E1 and slow dissociation (White* et al.* 2001).

## Discussion

It has been established that PVs, the small double-stranded DNA viruses, infect epithelial tissue of their mammalian hosts and replicate in mitotically active basal keratinocytes in which the viral genomes are maintained as a nuclear plasmid (episome). Generally, viruses causing acute infections usually transform the infected cell into a virus factory, eventually leading to cell death and release of the viral progeny (Lipsitch and O'Hagan 2007). However, PVs do not kill the infected cell. Instead, PV infections persist mostly for decades but are not very productive (Cubie 2013). The PVs in persistently infected mammalian host epithelia rely upon the host replication machinery for their replications. However, it still lacks mammalian cell systems and an animal model for the vegetative replication of HPVs. We previously demonstrated that replication of BPV-1 DNA was present in virion-infected *S. cerevisiae* cells. Here we report further that episomal replication of BPV-1 DNA persistently present in virions-infected *S. cerevisiae* by long-term culture (up to 108 days). The persistent replication of BPV-1 DNA in *S. cerevisiae* is attributed to several physiological factors that DNA replication machinery is generally conserved between humans and *S. cerevisiae* (Chattopadhyay* et al.* 2005) and the cellular origin recognition (ORC) complex is functionally conserved from yeast to mammalian cells (Vashee* et al.* 2001). Also, PVs possess autonomously replicating sequence-like elements that control viral DNA replication (Rogers* et al.* 2008). The consensus ARS sequence within the BPV-1 genome shares with yeast (Chuang and Kelly 1999).

In our experiments, we extracted the episomal BPV DNA from the long-term virion-infected yeast cultures (up to 108 days), proved the episomal replication of BPV-1 DNA in yeast cells by 1-DAGE and DNA Southern blot hybridization. It was interested to note that the episomal replication of the BPV-1 DNA could be divided into three stages, active replication, weak replication and stable replication over the three-time courses. The active replication of the BPV-1 DNA at the early stage (up to 16 or 23 days) could be that the replicating molecules had a high degree of superspiralization in at least part of the replicating genome, due to the intensive ongoing elongation of the synthesized DNA strand. The weak replication of the BPV-1 DNA in the middle stage (day 23–34 or 44) may be associated with that the replication of BPV-1 DNA could produce dramatic biochemical and structural changes, which might cause yeast cell damage. That may explain that the viral RNA transcription was very weak (Chen *et* *al.* 2021) and the virion-infected yeast cells grew slowly and unhealthy. At the late stage (day 45–108), the stable replication might be ascribed to the viral latency indicating that the BPV-1 virus was going to lie dormant within yeast cells, which was a typical characteristic shared by PVs (Araldi* et al.* 2017).

A previous study has reported that replication of BPV-1 genome in a subclone of 1ID13 mouse fibroblasts latently transformed with BPV-1 DNA occurs as a mixture of extrachromosomal circular monomers and oligomers (Schvartzman* et al.* 1990). However, no study has been reported to use real virions isolated from bovine papilloma to infect *S. cerevisiae* and mammalian cells and investigate the replication patterns and intermediates of the viral DNA. Previously, we observed that BPV-1 DNA partially digested with *Hin*dIII in the short-term BPV-1 virion-infected *S. cerevisiae* culture showed a single replication bubble, a typical replication pattern (Zhao and Frazer 2002a). In the present study, we investigated the replication patterns of BPV-1 DNA in BPV-1 infected *S. cerevisiae* culture over a long time period (82 days) by means of 2-DAGE and Southern blot hybridization. It has been reported that analysis of replication intermediates in HPV-18 might help to elucidate the complicated replication mechanisms of its viral DNA replication (Orav* et al.* 2015). Indeed, multiple replication intermediates have been detected in the present study. The detected replication intermediates including linear forms, single-stranded, double-stranded and heterogeneous double-stranded DNAs appeared to be the autonomous viral DNA strands, which were steady over the time period. The occurrences of the linear monomers, dimers, trimers, and higher oligomers occurred in *S. cerevisiae* may be well explained by a published study in mammalian cells (Schvartzman* et al.* 1990). Initiation of DNA replication of BPV-1 occurred near the centre of the *Eco*RI-*Bam*HI 5.6 kb fragment. The *Eco*RI-*Bam*HI 2.3 kb fragment replicated as a DNA molecule containing a termination site for DNA replication and also by means of a single fork traversing the fragment from one end to other. Thus, multiple copies of BPV-1 DNA occurred at a single site in a head-to-tail tandem array, a partial digestion with a restriction enzyme that cut the BPV-1 genome only once would generate a series of linear monomers, dimers, trimers, and higher oligomers (Schvartzman* et al.* 1990). The other replication intermediates such as conspicuous multimeric circular ssDNA, rolling circle replication and recombinant-dependent replication intermediates occurred in *S. cerevisiae* are very complex, which appear to be associated with the utilization of templates and their length (Erdmann, 2010; Erdmann* et al.* 2010). The Y-shaped replication intermediates containing branched molecules are probably due to a delayed fashion with a beginning of leftward replication (Belanger* et al.* 1996). Thus, the multiple replication intermediates detected in the present study may be ascribed to that BPV-1 has different replication modes in *S. cerevisiae*. Two replication modes: an ordered once-per-S-phase fashion and a random fashion have been reported for the replication of BPV-1 DNA in eukaryotic cells (Berg* et al.* 1986; Gilbert and Cohen 1987; Nallaseth and DePamphilis 1994; Ravnan* et al.* 1992).

In viruses such as hepatitis B virus (HBV) and hepadnaviruses, higher oligomer cccDNA is a key replication intermediate in the viral life cycle (Cheng* et al.* 2020; D'Arienzo* et al.* 2021; Li* et al.* 2017; Lucifora* et al.* 2017). The cccDNA persisted in the nucleus is a long-lived nucleosome-associated minichromosome, which is the main nuclear transcription template for producing all viral RNAs (Hong* et al.* 2017; Luo* et al.* 2020). Recently, covalently closed circular RNAs (cccRNAs) have been described in the human DNA tumor viruses Epstein-Barr virus (EBV) and Kaposi's sarcoma-associated herpesvirus (KSHV) (Toptan* et al.* 2018). In the present study, cccDNA was detected in the BPV-1 virion-infected *S. cerevisiae* cultures. However, the frequencies of cccDNA at the first stages (day 3–16) were significantly higher than those at the third stage (day 45–82) while the cccDNA appeared to be suppressed at the second stage (day 23–34) due to the unhealth growth of the virion-infected yeast cells. The high frequency of cccDNA at the first stage might directly contribute the high transcription of BPV-1 RNA (Chen* et al.* 2021). In addition, each time point at the third stage (day 45–82) that showed to have only one cccDNA occurred might suggest that replication of BPV-1 viral DNA was conserved to maintain its genomic DNA at low-copy numbers for the latent infection.

Recently, Toots and colleagues reported that several novel HR-HPV-specific inhibitors were identified to inhibit the HPV replication in the cells (Toots* et al.* 2017). These HR-HPV-specific inhibitors target Tdp1 and PARP1 leading to blocking HPV genome replication, colliding replication forks and eventually producing aberrant DNA replication intermediates (Toots* et al.* 2017). Thus, whether the identified HR-HPV-specific inhibitors could target BPV-1 L2 proteins, specifically the L2 CPP to prevent BPV genome replication leading to forming aberrant DNA replication intermediates is worthy of future study in BPV-1 virion-infected S. *cerevisiae*.

In conclusion, we have shown three episomal replication patterns of BPV-1 genomic DNA in virion-infected *S. cerevisiae* for long-term cultures (up to 108 days) by means of DAGE and Southern blot hybridization. BPV-1 genome could replicate as extrachromosomal dsDNA circular plasmids and maintain stable copy numbers at the late stage in the virion-infected yeast cells. Our data have revealed that multiple replication intermediates detected include the linear monomers, dimers and trimers, higher oligomers especially cccDNAs, single-stranded DNA, double-stranded DNA, heterogeneous double-stranded DNA, multimeric circular ssDNA, rolling circle replication and recombinant-dependent replication intermediates. The occurrence of these viral replication intermediates will improve our understanding of BPV-1 genomic DNA replication in *S. cerevisiae* system.

## Supplementary Information

Below is the link to the electronic supplementary material.

Supplementary file1 (PDF 281 kb)
