# Supplementary material for: Characterization of Episomal Replication of Bovine Papillomavirus Type 1 DNA in Long-Term Virion-Infected Saccharomyces Cerevisiae Culture
Source: Virol Sin. 2021 Aug 30;36(6):1492–502. doi: 10.1007/s12250-021-00439-y (PMC8692549; doi:10.1007/s12250-021-00439-y)
Supplement: Supplementary file 1 — Supplementary file1 (PDF 281 kb) [file 12250_2021_439_MOESM1_ESM.pdf]

VIROLOGICA SINICA

Electronic Supplementary Material

Characterization of Episomal Replication of Bovine Papillomavirus Type 1 DNA in Long-Term Virion-infected *Saccharomyces Cerevisiae* Culture

Quanmei Tu[1], Weixu Feng[2], Zhuo Chen[2], Qijia Li[1,2], Yu Zhao[1], Jun Chen[2], Pengfei Jiang[2], Xiangyang Xue[2], Lifang Zhang[2]✉, Kong-Nan Zhao[1,2,3]✉

1. Department of Obstetrics and Gynaecology, the Second Affiliated Hospital and Yuyin Children Hospital of Wenzhou Medical University, Wenzhou 325035, China
2. Institute of Molecular Virology and Immunology, Department of Microbiology and Immunology, School of Basic Medical Sciences, Wenzhou Medical University, Wenzhou 325035, China
3. Australian Institute for Bioengineering and Nanotechnology, the University of Queensland, St Lucia, Queensland 4067, Australia.

Supporting information to DOI: 10.1007/s12250-021-00439-y

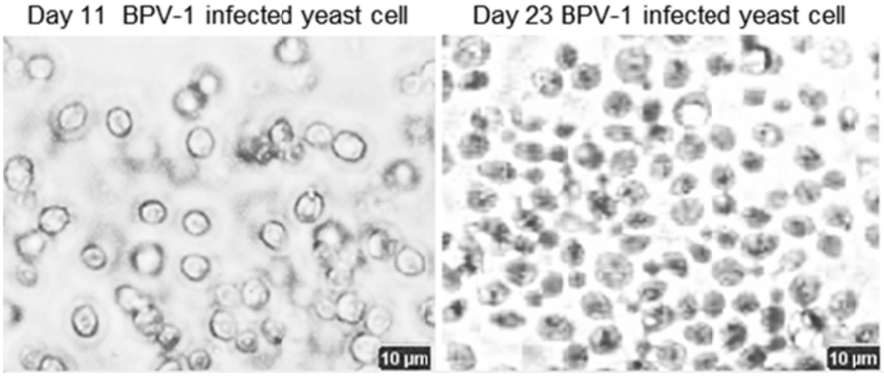

Figure S1. Morphology of BPV-1-infected *S. cerevisiae* cells in suspension culture with agitation at Day 11 compared with that at Day 23 visualized *under* a light microscope. Scale bars=10 µm.

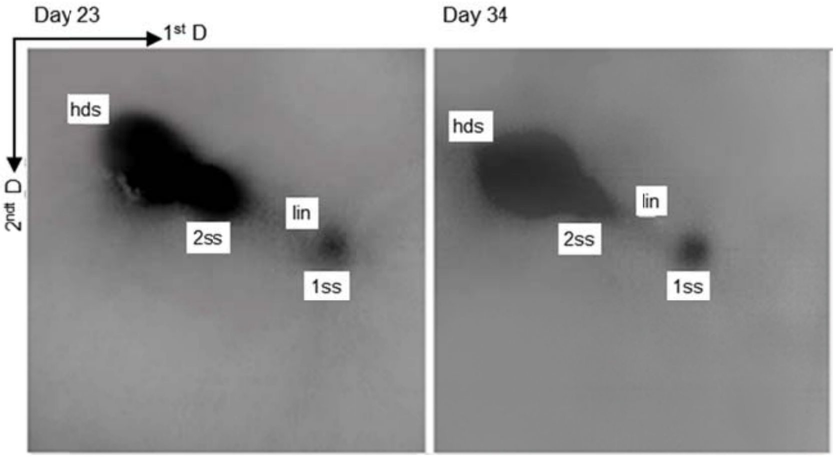

Figure S2. Analysis of viral DNA replication intermediates in BPV-1 virion-infected *S. cerevisiae* cells culture for 23 and 34 days by means of two-dimensional gel electrophoresis and Southern blot-hybridization analysis. Replication intermediates including single-stranded DNA (1ss), double-stranded DNA (2ss), heterogeneous double-stranded DNA (hds), and linear forms (lin) were present. But the oligomers such as covalently closed circles (CCC), open circles (OC), conspicuous multimeric circular ssDNA (cms) and rolling circle replication (RCR) were scarcely observed.
